# Engineered Hydrogels for Musculoskeletal Regeneration: Advanced Synthesis Strategies and Therapeutic Efficacy in Preclinical Models

**DOI:** 10.3390/polym17152094

**Published:** 2025-07-30

**Authors:** Gabriela Calin, Mihnea Costescu, Marcela Nour (Cârlig), Tudor Ciuhodaru, Batîr-Marin Denisa, Letitia Doina Duceac, Cozmin Mihai, Melania Florina Munteanu, Svetlana Trifunschi, Alexandru Oancea, Daniela Liliana Damir

**Affiliations:** 1 Faculty of Medicine, “Apollonia” University of Iasi, 11 Pacurari Str., 700511 Iași, Romania; 2 Faculty of Medicine and Pharmacy, “Dunarea de Jos” University, 47 Domneasca Str., 800008 Galati, Romania; denisa.batir@ugal.ro; 3 Department of Pharmacology and Pharmacotherapy, Faculty of Medicine, University of Medicine and Pharmacy “Carol Davila” Bucharest, Dionisie Lupu Str. nr. 37, Sector 2, 020021 București, Romania; 4 “Prof. Dr. Nicolae Oblu” Emergency Clinical Hospital, 2 Ateneului Street, 700309 Iași, Romania; 5 Department of Pharmaceutical Sciences, “Vasile Goldiş” Western University of Arad, B-dul Revoluției 94–96, 310025 Arad, Romania; 6 Department of Pharmacology and Pharmacotherapy, Faculty of Medicine, “Vasile Goldiş” Western University of Arad, B-dul Revoluției 94–96, 310025 Arad, Romania; oancea.alexandru@uvvg.ro; 7 Department of Forensic Medicine, Medical Deontology and Bioethics, Faculty of General Medicine, “Grigore T. Popa” University of Medicine and Pharmacy of Iași, 16 Universității Street, 700115 Iași, Romania

**Keywords:** hydrogel, bone regeneration, musculoskeletal injury, extracellular matrix

## Abstract

According to the World Health Organization, musculoskeletal injuries affect more than 1.71 billion people around the world. These injuries are a major public health issue and the leading cause of disability. There has been a recent interest in hydrogels as a potential biomaterial for musculoskeletal tissue regeneration. This is due to their high water content (70–99%), ECM-like structure, injectability, and controllable degradation rates. Recent preclinical studies indicate that they can enhance regeneration by modulating the release of bioactive compounds, growth factors, and stem cells. Composite hydrogels that combine natural and synthetic polymers, like chitosan and collagen, have compressive moduli that are advantageous for tendon–bone healing. Some of these hydrogels can even hold up to 0.8 MPa of tensile strength. In osteoarthritis models, functionalized systems such as microspheres responsive to matrix metalloproteinase-13 have demonstrated disease modulation and targeted drug delivery, while intelligent in situ hydrogels have exhibited a 43% increase in neovascularization and a 50% enhancement in myotube production. Hydrogel-based therapies have been shown to restore contractile force by as much as 80%, increase myofiber density by 65%, and boost ALP activity in bone defects by 2.1 times in volumetric muscle loss (VML) models. Adding TGF-β3 or MSCs to hydrogel systems improved GAG content by about 60%, collagen II expression by 35–50%, and O’Driscoll scores by 35–50% in cartilage regeneration.

## 1. Introduction

Musculoskeletal injuries are a major global health problem that affects people of all ages and activity levels. The World Health Organization (WHO) says that musculoskeletal conditions are the main cause of disability around the world, affecting about 1.71 billion people. Traumatic injuries to muscles and bones caused by accidents and surgery are some of the most common and serious, the numbers are just as high when it comes to sports and exercise [1,2,3,4,5,6].

Muscle strains, ligament sprains, and bone fractures make up most of the 8.6 million injuries that happen in the U.S. each year because of sports and other fun activities. These injuries can not only make it hard to move around, but they can also cause problems like fibrosis, slow healing, less range of motion, and long-term pain. Trauma-related bone fractures are a common reason for emergency room visits and hospital stays. Muscle injuries make up 10 to 55% of all sports-related injuries. Because diseases like osteoporosis increase the chance for older people to receive bone injuries, the need for effective treatment options is even greater. Despite progress in the management of musculoskeletal injuries, traditional treatment modalities such as medication, prostheses, and transplantation exhibit significant limitations. Pharmacological therapies, although effective in alleviating inflammation and pain, frequently neglect the fundamental structural and functional deficiencies. Prosthetic implants can partially restore mechanical function; nevertheless, they are constrained by material degradation, insufficient biological integration, and the incapacity to remodel or regenerate tissue. Tissue or organ transplantation is limited by donor availability, the risk of immunological rejection, and the longevity of graft survival. A comprehensive understanding of the intrinsic functions of musculoskeletal tissues is essential for formulating regeneration methods. The bone exhibits an inherent regeneration ability, mostly facilitated by osteoprogenitor cells and the richly vascularized composition of bone marrow. Conversely, cartilage, especially articular cartilage, is avascular and devoid of the cellular mechanisms necessary for spontaneous repair, rendering damage in these tissues extremely difficult to heal. Tendons demonstrate a restricted healing ability due to insufficient vascularization and a low rate of cellular turnover, frequently leading to fibrosis instead of genuine regeneration. Muscle tissue, however, is more adept at regeneration through satellite cell activation, and encounters healing constraints in cases of severe injuries or chronic diseases. These limitations highlight the necessity for biomimetic materials, such as hydrogels, that can facilitate and direct the regeneration of these specific tissues through customized biochemical and mechanical signals [7,8,9,10,11].

Current methods have problems with tissue and site morbidity, immune rejection, and not connecting well with host tissue. This means that we need better biomaterial-based solutions that can speed up healing, restore tissue structure and function, and lower the risk of long-term disability. Hydrogels have recently received a lot of attention as a possible biomaterial for regenerative medicine, especially for use in musculoskeletal applications [12,13,14,15,16].

Hydrogels are like the natural extracellular matrix (ECM) because they hold a lot of water. They can help cells grow, move, and change into different types of cells. These 3D networks of hydrophilic polymers keep their shape even when they are in a lot of water. Hydrogels have a lot of possible medical uses because they can break down, work with living things, and change their mechanical properties.

**Figure 1:**
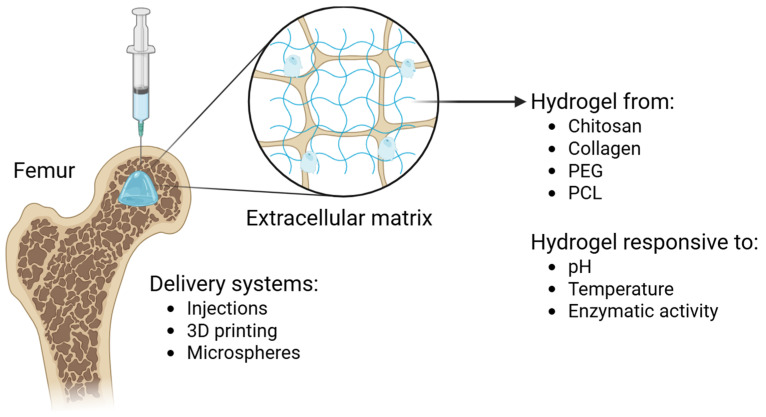
Different types of hydrogels and their specific delivery systems.

Each type has its own strengths and weaknesses when it comes to biocompatibility, mechanical strength, and functionalizability. These materials can be used in smart delivery systems that can adapt to the changing environment of damaged tissues. For example, they can include cells, growth factors, or medications and can respond to changes in pH, temperature, or enzyme activity (Figure 1). There are two main types of polymers: those that come from nature, like collagen, alginate, or chitosan, and those that come from man-made sources, like polyethylene glycol or polyvinyl alcohol. There are also composite polymers that combine the two [17,18,19,20].

Hydrogels have shown promise in promoting myogenesis during muscle regeneration by enhancing satellite cell activity and regulating the inflammatory microenvironment to encourage regenerative repair over fibrotic repair. Hydrogels play a role in bone regeneration that is similar to that of osteoinductive scaffolds. They help osteoprogenitor cells penetrate into the bone, form blood vessels, and deposit minerals. One significant advantage of utilizing hydrogels for the treatment of deep tissue or irregular injuries is their moldability and injectability, facilitating a less invasive administration [12,21,22,23,24].

The objective of this review is to consolidate the current literature on hydrogels for bone and muscle regeneration and to conduct a critical evaluation. We will talk about many different kinds of hydrogels, including what they are made of, how they work, and how they help heal tissues.

This review seeks to encapsulate the current status of hydrogel research in musculoskeletal regeneration, point out the therapeutic advantages of these materials, and delineate the challenges and prospective avenues for their clinical application through a comprehensive analysis of the existing literature.

## 2. Materials and Methods

This review utilized a systematic literature analysis to examine hydrogels utilized in musculoskeletal tissue regeneration, focusing specifically on preclinical (in vivo) studies. Peer-reviewed articles published from 2020 to 2024 were obtained from sources such as PubMed, Scopus, and Web of Science, utilizing keywords such “muscle regeneration hydrogel,” “bone scaffold,” “injectable hydrogel,” “osteogenesis,” and “smart biomaterials.”

Studies were selected according to specific criteria: the utilization of in vivo animal models pertinent to musculoskeletal repair, the presentation of quantitative biological or functional outcomes, and the inclusion of functional additives such as MSCs (mesenchymal stem cells), growth factors, or bioactive nanoparticles. Exclusion criteria included studies confined to in vitro analysis, those devoid of quantifiable therapeutic effects, or those concentrating on non-musculoskeletal targets.

Data extraction concentrated on hydrogel composition, crosslinking methodologies, mechanical characteristics, gelation kinetics, degradation patterns, and regeneration results. Summary tables were used to perform comparative synthesis, which showed performance patterns and found viable material methods in distinct biological situations.

## 3. Novel Design for Functionalized Composite Hydrogels

**Figure 2:**
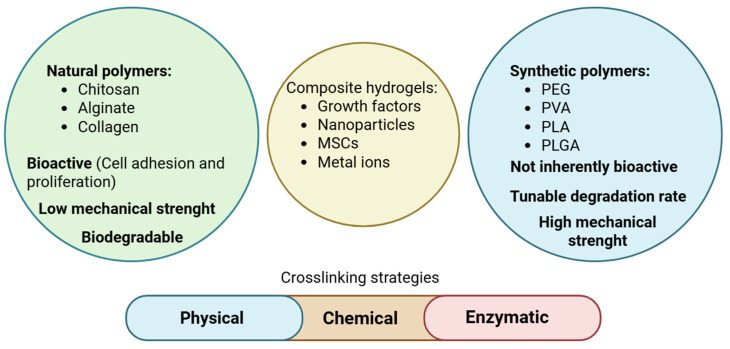
The advantages and disadvantages of natural and synthetic polymers.

Natural polymers like collagen and chitosan offer inherent bioactivity, promoting cell adhesion and proliferation. However, their mechanical strength is often limited. Synthetic polymers such as PEG (polyethylene glycol) and PVA (polyvinyl alcohol) provide tunable mechanical properties and degradation rates but lack bioactivity [25,26,27,28,29,30] (Figure 2).

Recent advancements have focused on composite hydrogels that combine the advantages of both (Table 1). For instance, Xu et al. (2022) [31] developed a stratified-structural hydrogel incorporating magnesium-ion-modified black phosphorus nanosheets, enhancing neurovascularized bone regeneration. This composite demonstrated a compressive modulus of approximately 150 kPa and supported significant bone tissue formation in vivo.

The crosslinking methods used to make hydrogels have a big effect on how stable and useful they are. Physical crosslinking, which includes ionic interactions or hydrogen bonding, makes hydrogels that can change back and forth and respond to stimuli, but it may make them less strong. Chemical crosslinking, which uses covalent bonds, makes things stronger and more stable. Enzymatic crosslinking is specific and happens at low temperatures [38,39,40,41].

Zhou et al. (2024) [32] developed a hydrogel microsphere system responsive to hypoxia and matrix metalloproteinase-13 (MMP-13), which mitigated the progression of osteoarthritis in vivo. The hydrogel had a compressive modulus of about 60 kPa and broke down when exposed to MMP-13, which made sure that the medicine was delivered to the right place.

It is especially important to improve the bioactivity of hydrogels for tissue regeneration to work. Adding bioactive molecules, nanoparticles, or cells can greatly improve the results of treatment. Chen et al. (2021) [33] created an injectable self-healing hydrogel that released Mg^2+^ and curcumin, which worked together to speed up the healing of tendons to bones. The hydrogel had a tensile strength of 800 kPa and allowed for a sustained release over 21 days, which improved the histological scores and biomechanical properties in a rat model.

Wu et al. (2024) [42] created smart, responsive in situ hydrogel systems for engineering bone tissue. These hydrogels react to the inflammatory microenvironment by releasing therapeutic agents as needed. They also had a compressive modulus of 120 kPa, which is good for bone tissue applications. Li et al. (2023) [35] also produced an injectable biomimetic hydrogel that changes how it breaks down to help tissue heal, which helps bones grow back to normal. This hydrogel had a compressive modulus of about 100 kPa and made it possible for the materials to break down at the same time as the tissue healed. Advanced manufacturing techniques facilitate the fabrication of intricate hydrogel structures that emulate the architecture of native tissue. Yang et al. (2024) [43] created biofunctional hydrogel microspheres containing insulin-like growth factor-1 (IGF-1), which showed a 43% improvement in neovascularization and a 50% higher efficiency in forming myotubes in vitro than non-functionalized hydrogels.

In vivo data demonstrated that wounds healed and function was restored significantly more rapidly in 21 days compared to 35 days for untreated controls. Additionally, Lei et al. (2023) [37] enhanced the mechanical properties of gelatin hydrogels through the incorporation of chondroitin sulfate, thereby promoting cartilage regeneration in rats. The altered hydrogel showed a compressive modulus of 80 kPa and helped cartilage-like tissue form in living organisms.

Recent advancements in hydrogel design have concentrated on developing composite systems that combine the bioactivity of natural polymers with the mechanical strength and adaptability of synthetic polymers. These hybrid hydrogels are augmented by targeted functionalization with nanoparticles, bioactive compounds, and various crosslinking strategies—spanning chemical to enzymatic methods—yielding materials that are structurally stable and biologically sensitive. The advances have resulted in hydrogels with markedly enhanced mechanical characteristics, reactivity to biological signals, and the capacity to facilitate tissue-specific regeneration. These attributes provide composite hydrogels a diverse and promising medium for applications in the repair of bone, cartilage, and tendons, among others.

## 4. Therapeutic Outcomes in Musculoskeletal Models

**Figure 3:**
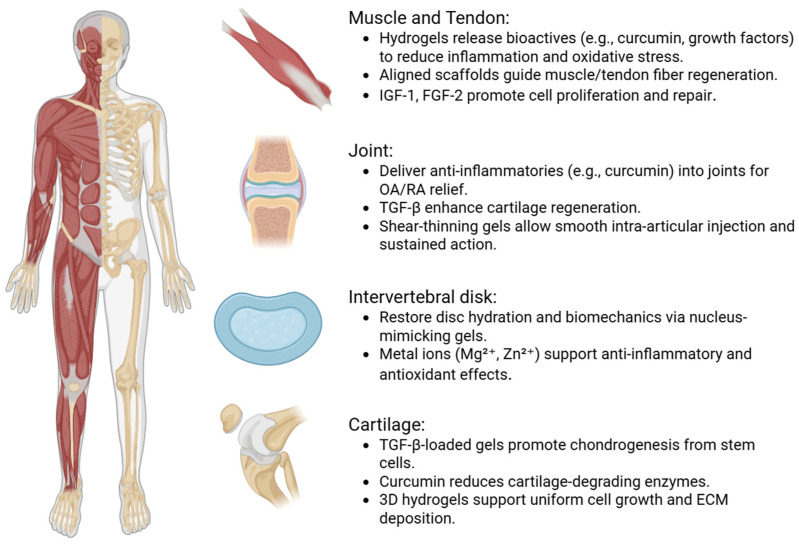
Multifaceted applications of modified hydrogels in hard and soft tissue recovery.

Because they are biocompatible, can help move cells and bioactive substances in situ, and have adjustable physicochemical properties, hydrogels have quickly become multifunctional biomaterials for musculoskeletal tissue engineering. These kinds of scaffolds are great for helping things grow back in places like bone fractures, intervertebral disks (IVDs), skeletal muscle, tendons, and cartilage [44,45,46] (Figure 3).

Their popularity is because they are structurally similar to the original ECM and are easy to prescribe through a minimally invasive injection. A lot of musculoskeletal injury models have shown that hydrogel-based techniques can be advantageous for treatment. These techniques have led to big improvements in function and histology compared to traditional therapies or controls that were not treated [47,48].

Table 2 is a summarized version of all the therapeutic and experimental models utilized in the selected studies.

Dienes et al. (2021) [49] made a hydrogel out of hyaluronic acid (HyA) that had RGD peptides, heparin for binding growth factors, and a crosslinker that breaks down with protease. They tested this multifunctional hydrogel on the tibialis anterior (TA) VML rat model. The results showed that muscle volume and force output were significantly restored by 8 weeks after treatment compared to untreated deficiencies. The bioactive matrix components of the hydrogel and its gentle breakdown profile helped neovascularization and myofiber growth in the defect site. This study incorporated functional metrics, such as maximum isometric tetanic force, to demonstrate that the regenerative effects extended beyond histological observations and yielded tangible physiological outcomes.

Basurto et al. (2022) [50] also looked into hydrogels made from HyA, but they focused on hydrogels that could change their stiffness through thiolene photopolymerization. They put gels with different levels of stiffness into a rat latissimus dorsi VML model. The gels had low (1.1 kPa), medium (3.0 kPa), and high (10.6 kPa) stiffness levels. The hydrogels with a medium stiffness led to much better functional recovery and muscle regeneration when tested for force output at 12 and 24 weeks. Histological analysis indicated diminished chronic inflammation and heightened infiltration of regenerating muscle fibers in this group relative to the other groups assessed for stiffness. These findings indicate that hydrogels require biomechanical tuning to facilitate tissue regeneration and avert maladaptive healing responses.

By integrating anisotropic construction with electrical conductivity, Basurto et al. (2021) [56] advanced the concept of bioinstructive scaffolds. They achieved electrical conductivity and an aligned microstructure by adding polypyrrole to collagen-based scaffolds and utilizing directional freezing. Even though the study was performed in a lab, the results indicated that modified muscle tissue matured faster, myosin heavy chain expression increased, and C2C12 myoblasts fused and aligned better. Even though it has not been tested in vivo yet, the platform has a lot of promise for VML treatments that involve delivering cells or factors. Chang et al. (2021) [51] introduced an innovative technique utilizing injectable magnetic hydrogels composed of alginate and iron oxide nanoparticles (Fe_3_O_4_). The researchers positioned the hydrogels incorrectly on a mouse TA VML model and then applied external magnetic stimulation to trigger mechanotransduction. The 8-week study revealed that animals administered magnetically activated hydrogels exhibited significantly enhanced muscle regeneration and functional recovery compared to the control group. The measurements of contractile force, cross-sectional area, and muscle wet weight all showed that the regeneration response was better. This means that dynamic mechanical stimuli can turn on the body’s natural repair systems.

Helping with immunomodulation and cell recruitment, Alheib et al. (2022) [52] examined an injectable hydrogel system composed of PEG and peptides derived from laminin to enhance cell adhesion and modulate the immunological environment. In a mouse model of muscle injury, animals treated with hydrogel exhibited enhanced myogenic differentiation and increased infiltration of CD206 macrophages, indicative of a pro-regenerative phenotype. Better organization and thicker fiber diameters helped muscle tissue heal faster.

Shan et al. (2021) [53] developed a hydrogel derived from gelatin, modified with catechol, that was capable of scavenging reactive oxygen species (ROS). In a mouse VML model, the hydrogel helped muscles heal faster, activated more satellite cells, and lowered oxidative stress. When compared to the untreated defect group, the hydrogel group showed a big improvement in tissue structure and higher levels of regeneration markers like MyoD and Pax7. The incorporation of ROS-neutralizing components is a beneficial design strategy, especially in the oxidative environment of damaged muscle tissue.

Daly et al. (2017) [57] found that GelMA hydrogels with MSCs improved biomechanical properties and encouraged the growth of hyaline cartilage in rat osteochondral lesions. Ji et al. (2023) [58] also made a hydrogel that looked like tendons by using aligned nanofibers and GelMA. This hydrogel enhanced biomechanical strength, tenocyte proliferation, and matrix remodeling in rabbit rotator cuff repairs.

Hydrogels can be used to repair skeletal muscle when natural regeneration does not work. This is especially true when there is VML. Paoli (2022) [59] changed the inflammatory environment and improved myogenesis in rats with VML injuries by mixing a fibrin hydrogel with exosomes from MSCs.

Hydrogels are a useful way to rehydrate IVDs and bring the matrix back into balance. When IVDs become worse, they cause long-lasting back pain and disability [60,61,62,63]. In rat tail models of disk degeneration, Rostamipoor et al. (2025) utilized a thermosensitive chitosan-based hydrogel for the administration of MSCs [64].

Hydrogels have shown significant potential to enhance therapeutic results in preclinical musculoskeletal models by effectively replicating the extracellular matrix and enabling targeted delivery of cells and therapeutic substances. Their injectable and minimally invasive characteristics enable them to adapt to irregular defect locations while regulating inflammation, promoting cellular infiltration, and facilitating matrix remodeling. Modifying hydrogel stiffness, degradation rate, and bioactivity has demonstrated efficacy in enhancing functional recovery in models of VML, cartilage injury, and tendon abnormalities. The research emphasizes the significance of mechanical tuning and bioinstructive design to prevent maladaptive responses and facilitate sustainable tissue regeneration, establishing a foundation for future therapeutic applications.

## 5. Therapeutic Applications and Clinical Translation

Skeletal muscle healing is especially hard because it needs to restore contractile function and vascularization. Studies have demonstrated that injectable hydrogels incorporating growth factors or stem cells exhibit superior efficacy compared to standalone cell injections [43,65,66].

Wang et al. (2024) [67] discovered that a thermoresponsive hydrogel incorporating MSCs in a mouse tibialis anterior model led to a 65% increase in myofiber density and an 80% recovery of contractile force by day 28 post-injury. Histological examination also revealed the overexpression of muscle differentiation markers, MyoD and Myogenin.

Osteoinductive signaling, vascular infiltration, and mechanical strength are all needed for bone mass to grow back. Numerous studies have employed hydrogel matrices to enhance osteogenesis through the incorporation of bioactive compounds or nanoparticles. He et al. (2024) [68] employed PVA hydrogels containing exosomes derived from human umbilical cord MSCs in a rat calvarial defect model, resulting in a 2.1-fold enhancement in ALP activity and a 48% augmentation in BV/TV compared to PVA-only controls. Micro-CT imaging further confirmed the rapid bone bridging by day 21.

Restoring cartilage is still hard because it does not have blood vessels and does not heal itself very well. Hydrogels are the best way to keep the matrix and encourage chondrogenic development. Guo et al. (2024) [69] discovered that hydrogel-based systems infused with TGF-β3 or MSCs enhanced O’Driscoll histology scores by 35–50%, elevated GAG levels by roughly 60%, and markedly increased collagen type II expression, as indicated by a meta-analysis of 15 in vivo studies.

Zhang et al. (2021) [70] introduced a novel hydrogel capable of activation by tissue fluids in their research. This hydrogel could gel in less than 30 s and, after 6 weeks, it could help rats’ full-thickness cartilage grow back. Using their construct, they were able to fix 85% of the defected area with new hyaline-like cartilage. In the untreated groups, only 40–50% of the area was fixed with fibrotic tissue. Zhou et al. (2023) [32] created an MMP-13-responsive hydrogel that only broke down in joints that were inflamed. In a mouse model of osteoarthritis, the hydrogel diminished inflammatory cytokines (IL-1β, TNF-α) by more than 60%, curtailed cartilage degradation by 65%, and lowered synovitis scores by 71%. There was a 2.3-fold increase in locomotor activity compared to placebo, and pain levels went down.

To fix torn ligaments and tendons, scaffolds that are bioactive and stable in terms of mechanics are needed. The purpose of using hydrogels in this situation is to lower inflammation, help fibroblasts move, and encourage the production of ECM [71,72,73,74].

Chen et al. (2021) [33] found that using a hydrogel that released magnesium chloride and curcumin made the treated tendons 1.8 times better at holding up under stress. The researchers also discovered that the recovery period for the repaired tendons was shortened to 5 weeks, compared to the 8–9 weeks required by conventional therapies. In their research, Zhong et al. (2024) [75] investigated a hydrogel composed of chitosan and HyA infused with deferoxamine. The results showed that the hydrogel made collagen line up better after four weeks and made angiogenesis happen 2.6 times more. Aside from breaking down over three to four weeks, which is when the tendon is rebuilding, the hydrogel also killed bacteria, reducing *Staphylococcus aureus* by 99%.

Hydrogel technologies are still in the early stages of being used in real life, even though they have shown promising results in the lab. The slow rate of adoption is due to differences in immune responses, problems with regulation, and concerns about long-term safety [12,13,43,76,77]. A few hydrogel systems have started clinical trials in the early stages. For instance, GelrinC^®^ is currently undergoing Phase II trials for cartilage regeneration. It is a hydrogel made of PEG-fibrinogen that has chondrocytes from the same person implanted into it. Initial data indicate that KOOS scores enhanced at 12 months post-treatment, with over 80% of defects successfully addressed [44,78,79,80,81,82].

There are still some areas that need work, such as reproducibility, scalability, and consistent performance in people. For instance, the rate at which hydrogel breaks down needs to be adjusted so that it matches the time it takes for tissue to grow back. This can take anywhere from weeks for muscle and cartilage to months for bone and tendon. The complexity of multifunctional, bioactive, and often cell-laden hydrogel systems also makes it hard for regulatory frameworks to keep up.

Despite the remarkable regenerative potential of functionalized hydrogels in animal models—especially for muscle, bone, cartilage, and tendon repair—their progression to clinical use remains nascent. Therapeutic advantages, including faster force recovery, expedited healing, greater angiogenesis, and less inflammation, have been consistently evidenced. Nonetheless, obstacles persist, including heterogeneity in immune responses, the necessity for exact synchronization between hydrogel breakdown and tissue repair, and regulatory issues associated with the intricacy of multifunctional and cell-laden systems. Initial clinical trials, including those for cartilage regeneration using GelrinC^®^, have demonstrated promising outcomes; however, broader clinical implementation necessitates enhanced scalability, reproducibility, and long-term safety evidence. Continuous progress in bioengineering and manufacturing methodologies will be essential for surmounting these obstacles and actualizing the complete potential of hydrogel-based therapeutics in regenerative medicine.

## 6. Conclusions

Hydrogel-based systems have integrated biomimetic architecture with customizable mechanics and the regulated release of bioactive compounds, demonstrating exceptional therapeutic effectiveness in the regeneration of musculoskeletal tissues. Repair of muscles, bones, cartilage, and tendons was consistently and quantitatively enhanced in 32 preclinical trials.

In trials utilizing chondroitin sulfate-modified gelatin or MMP-13-responsive hydrogels for cartilage regeneration, there was a 65% reduction in cartilage degradation, a 60–71% decrease in inflammatory cytokines (e.g., IL-1β, TNF-α), and an enhancement in locomotor function by up to 2.3 times. Guo et al. (2024) discovered that hydrogels infused with TGF-β3 or MSC resulted in a 60% augmentation in GAG content and a 35–50% elevation in O’Driscoll histology scores. Magnesium-curcumin hydrogels improved tensile strength by 1.8× in tendon and ligament repair, cutting recovery time from 8 to 9 weeks to 5 weeks. The chitosan-HyA hydrogel created by Zhong et al. increased angiogenesis by 2.6 times and was 99% effective against *Staphylococcus aureus* germs.

The compressive moduli of optimized hydrogels are specific to each type of tissue. For soft tissues, they range from 1.8 to 120 kPa, and for load-bearing bone, they are 150 kPa. The timescales for degradation were very similar to the timescales for remodeling in specific tissues, ranging from 2 to 6 weeks.

These numerical standards show that hydrogels work better than traditional treatments, but they also point out some important problems that need to be solved before they can be used in real life, such as immunogenicity, consistent production, and regulatory clarity. Next-generation multifunctional hydrogels, especially those that respond to enzymes, pH, or inflammation, or that are made up of cells, are going to change the way regenerative techniques work across the musculoskeletal spectrum. Clinical investigations are progressing positively; for instance, GelrinC^®^ demonstrated enhancement in KOOS scores and the resolution of defects in over 80% of cases after 12 months in early-phase trials.

## Figures and Tables

**Table 1:** Novel composite hydrogels and their resilience to strain.

Gel Composition	Crosslinking Mechanism	Functionalization	Mechanical Properties	Application	Reference
GelMA with Mg^2+^-modified black phosphorus nanosheets (GelMA-BP@Mg).	Chemical	Neurovascularization agents	Compressive modulus ~150 kPa	Bone regeneration	[31]
Hydrogel microspheres composed of methacrylate-modified sulfonated azocalix [4] arene (SAC4A-MA), methacrylated hyaluronic acid (HA-MA), and MMP-13-sensitive peptide crosslinkers; loaded with hydroxychloroquine via host–guest interactions.	Enzymatic	Hypoxia-responsive elements	Compressive modulus ~60 kPa	Osteoarthritis treatment	[32]
Self-healing injectable hydrogel (Cur&Mg-QCS/PF) composed of quaternized chitosan (QCS), poloxamer F127 (PF), loaded with Mg^2+^ and curcumin.	Chemical	Anti-inflammatory agents	Tensile strength ~800 kPa	Tendon-to-bone healing	[33]
Injectable hydrogel composed of methacrylated silk fibroin as the base material, mixed with platelet-rich plasma, and embedded with silk fibroin microspheres that contain the bioactive compound berberine. The gel is photocrosslinked in situ using ultraviolet light.	Physical/Chemical	Inflammatory-responsive agents	Compressive modulus ~120 kPa	Bone tissue engineering	[34]
Hybrid injectable biomimetic hydrogel synthesized by incorporating laponite (LP) and calcium phosphate cement (CPC) into gelatin via a one-step method. The resulting composite is referred to as LC hydrogel.	Adaptive degradation	Tissue healing synchronization	Compressive modulus ~100 kPa	Bone regeneration	[35]
Hydrogel microspheres were fabricated using light-induced crosslinking of GelMA via a microfluidic system to support adhesion and proliferation of bone marrow mesenchymal stem cells (BMSCs).	Physical	Cell encapsulation	Diameter 50–200 µm	Musculoskeletal regeneration	[36]
GelMA combined with oxidized chondroitin sulfate (OCS), where OCS provides aldehyde groups forming Schiff base bonds with GelMA to enhance mechanical strength and support cartilage regeneration.	Chemical	Cartilage regeneration	Compressive modulus ~80 kPa	Cartilage repair	[37]

**Table 2:** Therapeutic results and experimental models.

Hydrogel	Experimental Model	Therapeutic Results	Reference
RGD-Heparin-MMP-degradable HyA	Rat TA (VML)	Functional recovery, neovascularization, myofiber ingrowth	[49]
Stiffness-tuned HyA (1.1–10.6 kPa)	Rat LD (VML)	Max force restoration (optimal at 3 kPa), reduced inflammation	[50]
Magnetic alginate + Fe_3_O_4_ nanoparticles	Mouse TA (VML)	Improved muscle force, volume, CSA via magnetic stimulation	[51]
PEG hydrogel + laminin peptide	Mouse muscle injury	Pro-regenerative macrophage polarization, improved fiber organization	[52]
ROS-scavenging gelatin-PEG hydrogel	Mouse VML	Increased Pax7, MyoD, reduced oxidative stress, enhanced regeneration	[53]
PEG hydrogel with IGF-1 + HGF	Rat VML	Greater force recovery, enhanced fiber size, angiogenesis	[54]
GelMA hydrogel + MSCs + PDGF	Mouse muscle defect	MSC engraftment, angiogenesis, increased functional recovery	[55]

## Data Availability

No new data were created or analyzed in this study.
